# Multimodal Correlative Preclinical Whole Body Imaging and Segmentation

**DOI:** 10.1038/srep27940

**Published:** 2016-06-21

**Authors:** Ayelet Akselrod-Ballin, Hagit Dafni, Yoseph Addadi, Inbal Biton, Reut Avni, Yafit Brenner, Michal Neeman

**Affiliations:** 1 Department of Biological Regulation Weizmann Institute, Rehovot 76100 Israel; 2 Department of Veterinary Resources Weizmann Institute, Rehovot 76100 Israel; 3 Department of Biological Services Weizmann Institute, Rehovot 76100 Israel

## Abstract

Segmentation of anatomical structures and particularly abdominal organs is a fundamental problem for quantitative image analysis in preclinical research. This paper presents a novel approach for whole body segmentation of small animals in a multimodal setting of MR, CT and optical imaging. The algorithm integrates multiple imaging sequences into a machine learning framework, which generates supervoxels by an efficient hierarchical agglomerative strategy and utilizes multiple SVM-kNN classifiers each constrained by a heatmap prior region to compose the segmentation. We demonstrate results showing segmentation of mice images into several structures including the heart, lungs, liver, kidneys, stomach, vena cava, bladder, tumor, and skeleton structures. Experimental validation on a large set of mice and organs, indicated that our system outperforms alternative state of the art approaches. The system proposed can be generalized to various tissues and imaging modalities to produce automatic atlas-free segmentation, thereby enabling a wide range of applications in preclinical studies of small animal imaging.

Small animal *in-vivo* imaging plays an essential role in preclinical research aimed at understanding physiological processes as well as progression of disease and development of therapies. Imaging across multiple modalities, Magnetic Resonance (MR), computed tomography (CT), and optical imaging modalities, such as bioluminescence imaging (BLI) and fluorescence imaging (FLI) provide valuable complementary information for dynamic, structural, functional and molecular identification in preclinical studies^1^. Even within a single modality registration can be important, for correction of motion in longitudinal scans. Specifically for MRI, field dependent effects provide incentive for registration of data acquired at different field strength. Magnetic relaxation rates (R_1_ and R_2_) of many endogenous proteins and exogenously delivered contrast media show unique dependence on the magnetic field. Similarly, magnetic field affects contrast in arterial spin labeling (ASL) measurements of blood flow; chemical exchange saturation transfer (CEST) for detection of contrast agents and endogenous metabolites; blood oxygenation dependent (BOLD) contrast for detection of functional activity in response to hemodynamic challenges; and others. This magnetic field dependence creates a unique opportunity for another dimension of molecular imaging, but requires registration of MRI data acquired at different fields.

Segmentation of anatomical tissues is fundamental for accurate and robust multi-modal correlative imaging and quantitative analysis in preclinical research. The goal of image segmentation is the identification and delineation of coherent structures in the image. Manual delineation is difficult and time consuming and does not meet the accuracy, reproducibility and efficiency demands. Automatic segmentation of anatomical structures in medical images remains challenging due to issues such as noise, intensity non-uniformity, partial volume effect, shape complexity of the various structures, natural tissue intensity variations, and the overlap in intensity characteristics between the anatomical structures. Specifically, the difficulty in whole body small animal image segmentation stems from the small size of the structures, the variability in the structure types, and the need to compensate for cardiac and respiration motion artifacts.

## Segmentation of Whole body Small Animal Images

Computational algorithms for whole body small animal image segmentation are typically guided by an anatomical atlas reference image. Below we review prominent atlas based approaches designed for small animals.

One of the earlier whole body mouse atlases presented is the MOBY phantom. MOBY^2^ is a four-dimensional (4D) whole body anatomical mouse model including cardiac and respiratory motion models. The three-dimensional (3D) anatomy of the phantom was based on the Visible Mouse^3^ a 110 micron resolution magnetic resonance microscopy (MRM) volume image of a normal 16 weeks old male mouse data obtained from Duke University. The anatomical structures were manually segmented using the SURFdriver10 software and the organ phantom was built by fitting 3D non-uniform rational B-spline (NURBS) surfaces to each segmented structure.

The Digimouse atlas^4^ was generated by constructing a 3D volumetric whole body mouse atlas from coregistered x-ray CT and cryosection data of a normal nude male mouse. The images were acquired post mortem from a single mouse placed on a frame with fiducials and the segmentation was performed using interactive editing tools on a wide range of anatomical structures. The atlas includes the segmentation with coregistered PET, x-ray CT and cryosection images.

In^5^ the authors used three publicly available small-animal atlases (Sprague–Dawley rat, MOBY, and Digimouse), to build three articulated skeleton atlases. Major bone groups were manually segmented for each atlas skeleton. Then, a kinematic model for each atlas was built: each joint position was identified and the corresponding degrees of freedom were specified. Similarly, in^6^,^7^,^8^ an atlas based registration method was presented warping the digimouse atlas to a surface of optical imaging data. The authors in^9^ proposed a non-rigid whole body skeleton registration for mice, based on 3D shape context model of point based surface registration.

Baiker *et al*.^10^,^11^ presented an atlas based approach for whole body segmentation of mice from low-contrast Micro-CT acquired *in-vivo*. The authors combine atlas-based registration utilizing high-contrast organs in Micro-CT (skeleton, lungs and skin) and then soft tissue approximation. The registration involves global alignment followed by registration of the individual bones as well as the lungs of the MOBY atlas by defining a hierarchical tree model. The abdominal organs are interpolated from the atlas to the subject domain based on a Thin-Plate-Spline (TPS) approximation^12^. Their approach was validated for registration on 26 non-contrast enhanced Micro-CT mice and the organ interpolation was evaluated using 15 contrast enhanced Micro-CT images. This approach was later applied by^13^ for super-resolution reconstruction of MRI in whole-body mouse data in studies of tumor metastases. Recently^14^,^15^ developed an automatic statistical atlas (multi-subject) approach to registration of micro-CT images in mice. The statistical atlas shape model registration first registers high contrast organs, and then estimates low contrast organs based on the first set of registered organs. To register the high contrast organs, the authors utilized a 2D-registration-back-projection scheme that deforms the 3D atlas based on an anterior–posterior X-ray projection and a lateral optical photo of the mouse silhouette.

Atlas guided approaches are a useful tool for medical image segmentation when a standard atlas is available^16^. Yet, different studies require significantly diverse data acquisition protocols. Differences may occur in terms of strain of the mouse, positioning variability, size, gender, age, the type of organs defined, resolution and modalities involved. The segmentation is limited by the ability of the atlas to represent the type of images under consideration. Specifically, all the previous atlases are typically based on normal male mice data^4^. Additionally, while high accuracy has been shown with skeleton and bones, segmentation accuracy for abdominal organs has been quite limited due to the poor soft tissue contrast in micro-CT^17^. The approach we present is utilized with data acquired *in-vivo*, including abdominal organs and can be adapted to a wide range of modalities and structures making it useful for automatic segmentation, future atlas constructions. The ability to automatically segment the data without the need for a reference atlas enables the study of organs and tissues that change between animals or between time points. Important examples include pathologies, such as growth of tumors, as well as physiological remodeling of tissues: for example the ovarian cycle and its effects on the uterus and ovaries, embryo implantation and multi fetal pregnancy.

## Related Segmentation methods

Superpixel segmentation is a common preprocessing step in many computer vision applications. The term “superpixel”/“supervoxel” refers to grouping the image pixels/voxels, into uniform atomic regions enclosed in structures^18^. The main advantage is the complexity reduction due to the smaller number of primitives compared to the number of pixels. Recent superpixel algorithm examples include reliance on meanshift^19^, minimum spanning trees^20^ k means clustering^21^, gradient ascent based approaches^22^,^23^,^24^ or graph representation of the^25^,^26^.

Graph-based segmentation techniques represent the image as a graph with vertices, edges and weights. Local variation by^20^ is a graph based approach such that each superpixel is the minimum spanning tree of pixel elements with complexity of *O*(*nlogn*) where *n* is the number of voxels. Shi and Malik^26^, proposed a spectral analysis technique to partition the image graph, based on finding the optimal cut. They introduced the normalized cuts criterion, which examines the similarities between neighboring pixels taking into account self-similarities of regions being separated. Once the two sets yielding the optimal cut have been found the procedure can be repeated iteratively until a desired number of superpixels is reached^24^. The complexity of the normalized-cut is *O*(*n*^*3/2*^) . The segmentation by weighted aggregation (SWA)^27^ approach is an effective acceleration of the normalized cut approach inspired by algebraic multigrid^28^. Alpert *et al*.^29^ further developed a probabilistic formulation based on the SWA algorithm. Additionally^30^, incorporated a Bayesian model into the calculation of affinities of the SWA algorithm and applied the model to the problem of brain tumors and edema segmentation in MR data. Applying this approach to different structures would require designing different parametric models and it is not clear whether the unified probabilistic framework would scale up to the case of multiclass whole body segmentation with a wide range of shapes and appearances.

Machine learning methods have been extensively used for medical image segmentation^31^,^32^ and can be divided to parametric and nonparametric methods. Parametric methods include for example decision trees^33^ artificial neural networks^34^ and Support Vector Machines (SVM). SVM’s were introduced by^35^ and have obtained successful performance in many pattern recognition applications. Nonparametric methods, do not require learning parameters and instead rely on the data directly^36^. The nearest neighbor (NN) method is a common nonparametric algorithm in which given a collection of training objects and a query object, builds a data structure which classifies according to the training object that is most similar to the query^37^. The simplicity of the NN algorithm avoids the common parameter overfitting problem and allows working with any distance function and number of classes.

In this work we propose a machine learning approach to compose the supervoxels into segmented structures. The use of machine learning is justified because of its ability to learn robust models with a small set of labeled images. Our method incorporates a bottom-up hierarchical agglomerative strategy based on pixel similarities in a graph-based approach. Although any supervoxel technique can be considered, we adapt SWA as a rapid and effective way to obtain a full hierarchy of superpixels in *O*(*n*). Then inspired by the work of^38^ and motivated by physiological and computational models of humans^39^, we utilize a joint SVM-kNN approach constrained to multiple heatmap bounding box (BB) regions. The key idea of constrained SVM-kNN is that a query is compared to all training examples in the region to obtain the “k-nearest neighbors” (kNN), and then a refined classification of SVM is performed amongst these neighbors to classify the query. The integration of the two methods, which operate on the same distance measure allows combining the advantages of both methods, avoiding the kNN problem of high variance (in bias-variance decomposition) and improving the time optimization of SVM.

## Novelty of this contribution

In previous work, a multiscale method for automated segmentation of multiple sclerosis in brain MR images that extended the Segmentation by Weighted Aggregation (SWA) to 3D and multi-modal data was developed^40^. Building on that study we report here a novel application for multimodal body imaging, overcoming multiple challenges relative to previous studies:

- First, the study focuses on small animals and abdominal organs and demonstrates results in a large multi-modal experiment combining information from MR, CT and optical imaging for small animals. To date advanced imaging and surgical technologies are becoming available in the case of clinical data for humans^41^, nevertheless existing segmentation methods developed for small animals are commonly designed for specific organ, modalities, or applications.
- Second, the algorithm does not rely on atlases. Existing approaches to whole body segmentation are based on atlases and are therefore usually tuned for specific set of structures, mouse models, mouse holders, gender, and modalities from which they were constructed. In contrast, the algorithm utilizes a heatmap based constraint relaxing the methods dependence on accurate registration of atlases while keeping the location prior knowledge and can therefore be applicable to wide range of problems. Moreover, studies producing atlases can benefit from our approach by employing an automatic segmentation approach rather than an interactive approach.
- Third, the system starts with an efficient supervoxel extraction based on SWA, where only intensity and location feature statistics are accumulated followed by multiple SVM-kNN classifiers each constrained to high probability regions which are later composed to obtain the segments structures.

In summary, our approach makes the following contributions:

- The development of an automatic segmentation method for a wide range of anatomical structures and ovarian tumors incorporating a machine learning strategy to graph based supervoxels. To our knowledge there are few reports of methods with such a wide variety of structures and modalities that have been extensively validated for preclinical research (See Table 1).
- Propose an effective 3D registration approach of BLI to MR based on point set registration of the whole body mouse surface utilizing a Gaussian mixture models (GMM) formulation.
- Advance the state of the art significantly in (1) providing a novel *in-vivo* multimodal small animal benchmark experimental data set. (2) Obtaining improved accuracy results on a wide range of anatomies verified on an extensive dataset.

The remainder of this paper is organized as follows: section 2 describes the main steps of the system and segmentation algorithm. Section 3 provides experimental results obtained by our approach in comparison to other approaches. Section 4 summarizes our conclusions and discusses future extensions of our approach.

## Material and Methods

Animal experiments were approved by the Weizmann Institutional Animal Care and Use Committee and all experiments were performed in accordance with the approved guidelines.

**Figure 1:**
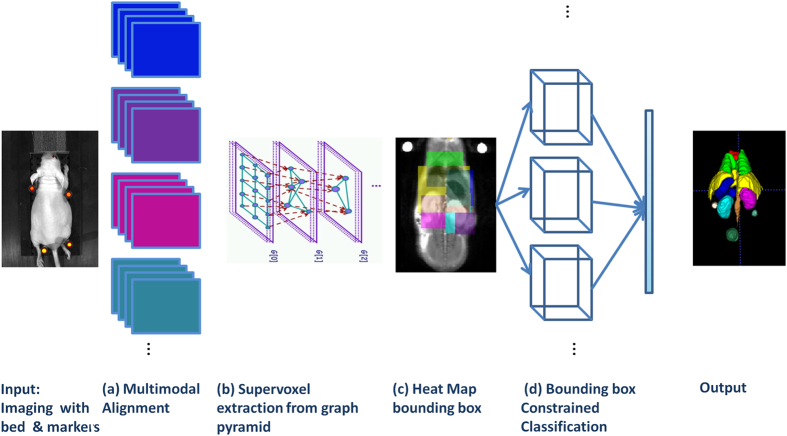
System Outline. Input: Mouse is placed on bed and imaged sequentially across modalities. (**a**) The multimodal data obtained is aligned. (**b**) Graph pyramid construction. The image illustrates three graph levels above the input data blocks. (**c**) Regional bounding box based on structure prior map. (**d**) Supervoxels are classified to obtain the output of structure segmentation.

The algorithm proposed is composed of several key steps: First, after performing the multi modal imaging with the designed bed and markers we register all imaging modalities to one pre-selected channel (in our experiments the MR 9.4T T2w). In the second step we generate the graph pyramid based on the multi-modal data and extract supervoxels from intermediate levels of the multi-scale graph. In the third step a set of bounding boxes are determined automatically for each structure region by defining a coordinate systems on the mouse body and generating an average heat map per structure based on a the training set coordinates. In the fourth step, we apply a machine learning approach to classify the supervoxels in each heat map region. Finally, we compose the results to obtain the segmentation output. Figure 1, shows a schematic representation of the algorithm.

**Figure 2:**
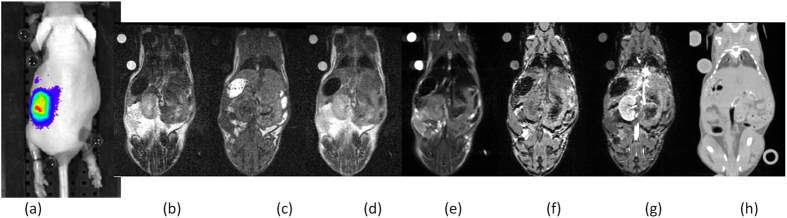
Imaging Acquisition. Illustration of the experiment flow and sequences acquired and processed by the algorithm: (**a**) Optical imaging BLI; (**b**) MR 1T T2w; (**c**) MR 1T T1w; (**d**) MR 1T T2w; (**e**) MR 9.4T T2w (different TR, TE); (**f**) MR 9.4T T1w; (**g**) MR 9.4T T1w + GdDPTA; (**h**) Micro-CT. The example demonstrates the benefit of multimodal imaging.

The approach was validated using twenty datasets of mice bearing tumor (14, 6 datasets of larger and smaller mice respectively). Mice were anesthetized and placed on a custom-made cross-modality bed loaded with fluorescent markers, and imaged sequentially with high field MRI (9.4T Bruker), low field MRI (1T Aspect), optical imaging (IVIS, Caliper) for bioluminescence (BLI), and micro-CT (Tomoscope). Figure 2, illustrates the sequences acquired and used by the system.

### Data acquisition and preparation

#### Bed manufacturing

**Figure 3:**
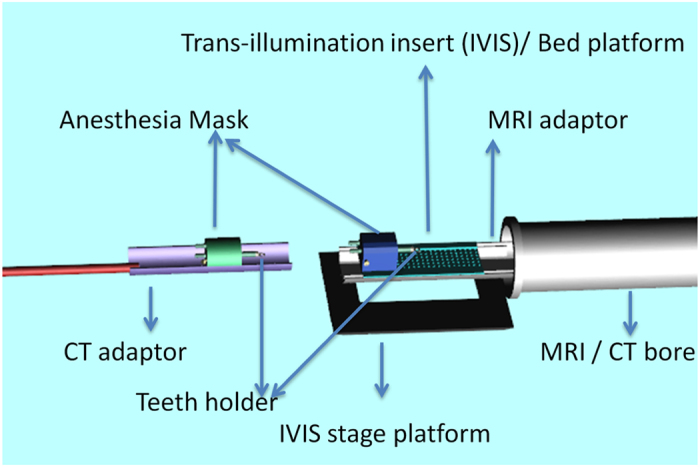
Animal Bed Design.

An animal bed for multimodal imaging was specifically designed and manufactured for this project (see Fig. 3). Design guidelines were based on the specific requirements of each of the imaging modalities. Bed was made out of black plastic in order to minimize optical reflection and in order to be MR compatible. Bed size and design was matched to the available insert in the IVIS spectrum stag, holes were created at the bottom of the bed at the exact pattern found in the IVIS stage in order to enable the use of trans-illumination. Same holes were used for insertion of fiducial markers and insertion of small rubber bands used to fix the animal to the bed. Fiducial markers were created by small vials, with black walls and open top, positioned vertically next to the body of the mice. Vials were filled with a solution of 10 mg/ml dextran FITC (sigma) that was detectable by epi-fluorescence, MR and CT imaging. Finally, mice were secured to a portal bed using small rubber bands on limbs and teeth holder and imaged sequentially by different modalities while positioned on bed with markers detectable by CT, IVIS and MRI to ensure accurate alignment between modalities.

#### Animal preparation for imaging

All experiments were approved by the Institutional Animal Care and Use Committee of the Weizmann Institute of Science. Animals were purchased from Harlan Laboratories Ltd (Jerusalem, Israel). Orthotopic ovarian tumor model was studied in female CD-1 nude mice 2 weeks after initiation of tumor. To generate the tumor, 6–8 weeks old mice were anesthetized with ketamine (100 mg/kg intraperitoneally; Fort Dodge Animal Health) and xylazin (20 mg/kg intraperitoneally; XYL-M2, V.M.D., Arendonk) and 0.5 × 10^6^ ES2-Luc-DsRed cells were injected into the bursa of the ovary through a small 2–3 mm skin incision in the back of the animal. The incision was then sutured and the animals were allowed to recover on a warmed pad. ES2-Luc-DsRed ovarian carcinoma cells express luciferase (Luc) and red fluorescent protein (DsRed) that allow BLI and fluorescent imaging^42^.

On the day of imaging, the animals were anesthetized as described above, positioned on the specially designed bed and the tail vein was catheterized. In between imaging modalities, the depth of anesthesia was evaluated and additional half-dose of anesthetics was added subcutaneously if needed. Transferring between imaging modalities was done while the animal was kept in the bed and with taking care not to change the position of the animal. Imaging order was: BLI, MRI 9.4T, MRI 1T and CT.

#### *In vivo* MR image acquisition on 9.4T magnet

MRI experiments at 9.4T were performed with a horizontal bore spectrometer (Bruker Biospec; Karlsruhe, Germany). A quadrature volume coil, with 72 mm inner diameter and a homogeneous RF field of 100 mm along the axis of the magnetic field, was used for both RF transmit and receive.

Coronal T_2_-weighted fast spin-echo images were acquired using RARE sequence with the following parameters: TR = 3000 ms; effective TE = 40 ms; slice thickness = 1.0 mm; inter slice gap = 0.1 mm; FOV = 6.4 × 6.4 cm^2^; matrix 256 × 128, zero filled to 256 × 256; rapid acquisition with relaxation enhancement (RARE) factor = 8; number of slices = 24; number of averages = 4.

Coronal 3D T_1_-weigthed gradient-echo images were acquired using MDEFT sequence with the following parameters: pulse flip angle = 15°; TR = 10 ms; TE = 3 ms; slice thickness = 0.5 mm; no inter slice gap; FOV = 6.4 × 6.4 cm^2^; matrix 256 × 128, zero filled to 256 × 256; number of slices = 48; number of averages = 2. After the first acquisition of the T_1_-weigthed image, contrast material BSA-GdDTPA^43^ was injected through the tail vain catheter and imaging was repeated 3 times.

#### *In vivo* MR image acquisition on 1T magnet

MRI experiments at 1T were performed with a permanent magnet (Aspect, Israel).

Coronal T_2_-weighted fast spin-echo images were acquired using T2-FSE sequence with the following parameters: TR = 2791 (or 4335) ms; TE = 80 (or 40) ms; slice thickness = 1.0 mm; inter slice gap = 0.1 mm; FOV = 6.4 × 6.4 cm^2^; matrix 256 × 180, [zero filled to 256 × 256]; number of slices = 20; number of averages = 2.

Coronal 3D T_1_-weigthed gradient-echo images were acquired using GRE-SP sequence with the following parameters: pulse flip angle = 35°; TR = 10 ms; TE = 2.6 ms; slice thickness = 0.5 mm; no inter slice gap; FOV = 6.4 × 6.4 cm^2^; matrix 256 × 256; number of slices = 48; number of averages = 2.

#### Optical imaging

Tumor development was followed using bioluminescence imaging (BLI) on the IVIS spectrum imaging system (Caliper Life Sciences). For BLI imaging mice were given an intra-peritoneal injection of 1.5 mg of D-luciferin (Caliper Life Sciences), sequential imaging iterations of 1 min exposure were performed until signal reached maximal plateau (about 15 min post D-luciferin IP injection). A single mouse was imaged at a time, Signal was acquired in BLI mode for 60 sec’ (no excitation and open emission filter) field of view of 12.6 cm’, Field stop and binning were selected according to signal in order to enable maximal signal without saturation.

#### *In vivo* micro-CT

The set of mice were scanned using a micro-CT device TomoScope^®^ 30S Duo scanner (CT Imaging, Germany) equipped with two source-detector systems. The operation voltages of both tubes were 40 kV. The integration time of protocols was 90 ms (360 rotation) for 3 cm length and axial images were obtained at an isotropic resolution of 80 μ. Due to the maximum length limit, to cover the whole mouse body, imaging was performed in two parts with overlapping area and then all slices merged to one dataset representing the entire ROI. The radiation dose for each mouse was 2.2 Gy.

#### Image Preprocessing: Registration and Manual segmentation

**Figure 4:**
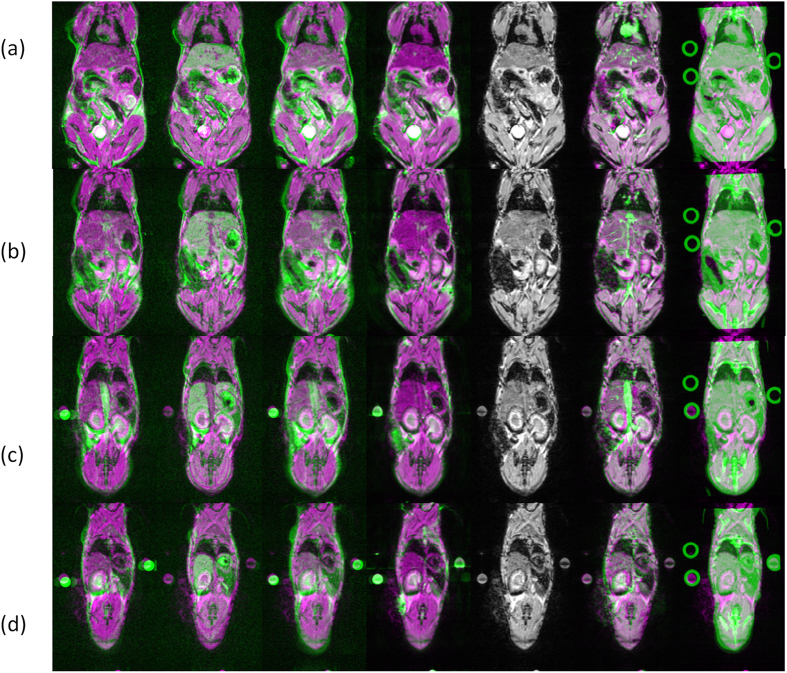
Alignment illustration. The columns present 2D slices of all MR and CT moving sequences post registration overlaid on the fixed reference (MR 9.4T T2w sequence). The rows (**a–d**) correspond to a different 2D axial slice. Each row includes from left to right MR 1T T2w , MR 1T T1w; MR 1T T2w (different TR, TE); MR 9.4T T2w; MR 9.4T T1w; MR 9.4T T1w + GdDPTA; Micro-CT (presented in green) and the fixed sequence (presented in magenta). The image was produced with the matlab imshowpair function. Since every sequence has a different intensity profile the amount of green in each anatomical structures varies between the sequences/columns. The column of the reference MR 9.4T T2w is the only grey image.

The data set for the experiment includes multi-modal 3D images of twenty mice and their manual segmentation (will be available on our group site). For each mouse, all 3D images were brought into the same frame of reference of the T1w channel on the 9.4T magnet. The advantage of the selected mouse holder is the ability to transport a small size anaesthetized mouse on bed from one scanner to the other, allowing us to make simple rigid body assumptions for inter-modality images. Spatial registration of MR channels was performed using SPM8 software (http://www.fil.ion.ucl.ac.uk/spm)^44^, The registration of the CT was performed using ITK software. Several cases in which the registration did not succeed were manually corrected based on the circular fiducial markers. Figure 4, demonstrates the registration results.

The manual segmentation of MR mice data was performed using the itksnap software (www.itksnap.org)^45^, based on the MR soft tissue contrast in all organs. The manual ground truth segmentation included nine structures: heart, lungs, liver, stomach, left kidney, right kidney, tumor, vena cava and bladder (Fig. 1; See Materials and Methods for illustration). Manual segmentation for four additional classes was generated in the CT data by manually assigning bright supervoxels (above a predefined threshold) to one of the following categories (upper limbs, ribs, spine and lower limbs).

#### Segmentation methodology

In this section, we first briefly explain the SWA approach for generating supervoxels together with our extensions^40^ (we refer the reader to the appendix for a detailed formulation) and then we explain the machine learning formulation.

#### Supervoxel extraction

The SWA algorithm uses a graph representation of the images and constructs a pyramid of graphs, which adaptively represents increasingly larger aggregates of voxels of similar properties. The nodes and the edge values of the initial graph are the voxels of the given images and similarity measures between neighboring voxels, respectively. The algorithm recursively coarsens the graph, level after level, by softly aggregating several similar nodes of a finer level into a single node of the next coarser level. The edges of the coarser graph are based on statistical features which are computed throughout the coarsening process for each aggregate. At each coarse scale there are about half as many nodes as in the next finer scale. The algorithm produces a hierarchy of supervoxels, each larger segment possibly containing several smaller ones.

**Figure 5:**
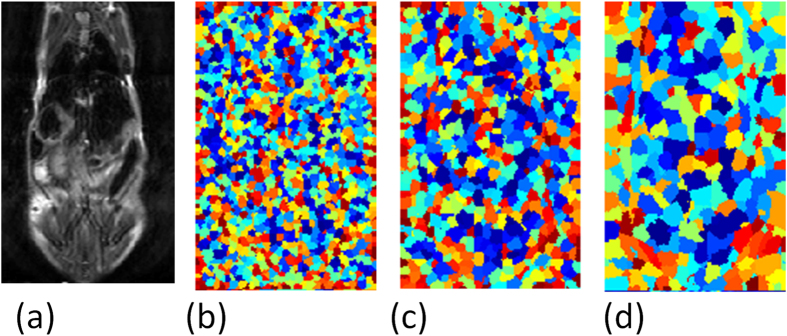
Supervoxel Illustration (**a–c**): Typical supervoxels extracted from three intermediate scales, (**d**) corresponding slice of original image.

The limitation of the hierarchical supervoxel algorithms is that there is no guarantee that a structure will appear at any level of the tree. Commonly, fine levels lead to over segmentation (more segments than structures) and coarse levels lead to under segmentation (missing structures). Thus supervoxel extraction from intermediate levels of the pyramid aims to overcome this limitation. The intermediate levels allow supervoxels to gather enough statistics before they merge with other structures and are determined based on the volume characteristics of the anatomical structures (scales 4–6 of ~14). Figure 5, illustrates supervoxels extracted in intermediate levels.

#### Automatic weight selection

Generally segmentation results may depend critically on the proper assignment of parameter values. In cases where multispectral data is available one of the difficulties lies in integrating the information into a combined similarity measure. Thus, in our approach the weight coefficient for each channel are determined automatically based on the gradient dominance of every channel. Such that given *m* = 6 channels 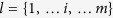, we set the weights based on the average gradient proportion along the surface border of the manual segmentation for all structures. Two randomly chosen data sets were used to determine the weight coefficient [0.14, 0.14, 0.13, 0.13, 0.23, 0.25] and these sets were not used later in either the training or testing experiments.

#### Heatmap Computation

The heatmap for each mouse, is constructed based on affine co-registrations of the manually labeled training set data to the test subject. First, the training sets are aligned with the test set and then the prior map is created by voxel-wise averaging of the tissue structures over the manually labeled training data sets. The probability function is formed by the frequency that each structure occurred at a voxel across the training sets. The map obtained, represents the prior probability of each voxel in the test set to belong to a particular structure. Due to the risk of biases of non-rigid deformation between mice the structure region map is defined by the bounding box (BB) of pixels with positive frequency.

#### Machine Learning Model

The unsupervised hierarchical supervoxel extraction process is integrated with an SVM-kNN heatmap constrained learning model. The heatmap BB constraint yields a fast coarse categorization followed by the kNN model obtaining a smaller focused NN set of training examples and completed by the SVM executing a fine discrimination of the test supervoxels.

Assuming a training set of q supervoxels, 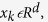 *k* = 1, …*q* extracted from intermediate levels of the pyramid, each represented by a *d* dimensional feature vector, the features are first normalized to zero mean and unit variance. The classifier is trained in a leave-one-out cross-validation strategy on the two sets of mice. The BB is used in the machine-learning model to split the training and testing supervoxels to regions. BB may overlap and class labels are not suppressed outside the regional BB. Training is performed on a multi-class problem using nine categories each representing a tissue class. Finally, when testing a new supervoxel all the BB decisions are merged by a voting scheme.

Our method trains an SVM on the k-nearest neighbors and directly obtains local decision boundary. The parameter k = 100 is selected according to best performance. SVMs attempt to find a separating hyperplane which maximizes the margins between the classes while minimizing the error on the training set with a cost depending on the number of misclassifications (C = 2^8^ is the penalty parameter for misclassification on the training data). Typically SVM maps the data from the original feature space to a higher dimensional space. Our experiments were performed utilizing the LIBSVM software package (available at http://www.csie.ntu.edu.tw/~cjlin/libsvm)^46^, with a non-linear SVM using a radial basis function kernel.
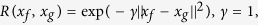


Summary of Machine Learning Steps

- Split training/testing supervoxels according to heatmap BB generated.
- For each BB, and all unseen test samples:
  1. Select the k nearest neighbors based on pairwise distance to training set.
  2. If all k neighbors are of the same class, label the query and exit;
  3. Else,
    - train multiclass SVM with kernel matrix only with kNN supervoxels.
    - Use the resulting classifier to label the query.
- Merge all BB-classifier labels by voting scheme, for all test samples.

#### BLI analysis

Optical imaging modalities such as Bioluminescence imaging (BLI) are widely used *in-vivo* to monitor biochemistry with high sensitivity specifically to follow tumors, albeit BLI does not provide anatomical information and therefore it is commonly fused with high resolution micro-CT images. In this study we focus on segmenting soft tissue organs, taking advantage of MRI’s excellent tissue contrast, thus all the modalities are aligned with an MR channel as the reference space.

To fuse the BLI data with the MR data we first perform three-dimensional (3D) reconstruction of the luminescent light source distribution by the Living Image software which is based on a diffuse tomography model (DLIT). We then extract points representing the skin surface in MR and BLI data automatically. The skin surface of the MR reference is detected in the coarse scale of the automated supervoxel hierarchy. Finally a point based registration approach^47^ based on a GMM formulation is applied minimizing the distance measure between the two corresponding mixtures.

**Figure 6:**
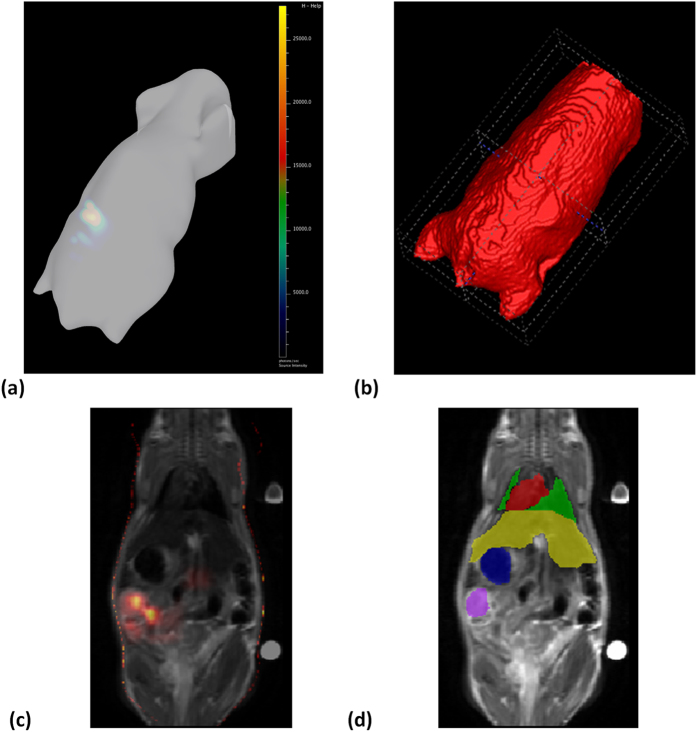
Registration of bioluminescence imaging to MRI. (**a**) 3D reconstruction of bioluminescence imaging (IVIS Spectrum). (**b**) MR whole body coarse segmentation. (**c**) Bioluminescence superimposed on MR after registration. (**d**) GT segmentation (heart in red, lungs in green, liver in yellow, stomach in blue, tumor in purple).

The combined use of MRI and CT with optical imaging allows integration of anatomical, functional, and metabolic information enhancing tumor identification. Figure 6 shows the results of alignment of BLI and MR and provides visualization of the GT tumor overlap with the positive BLI signal. However, the BLI imaging technique is not accurate enough for tumor localization. Namely, currently inclusion of the BLI signal in the segmentation could lead to disagreement between the automatic and manual segmentation with many false positives (FP) and false negatives (FN) in tumor delineation. The limitation stems from the low resolution due to light scattering in the tissue.

## Results

### Validation

**Figure 7:**
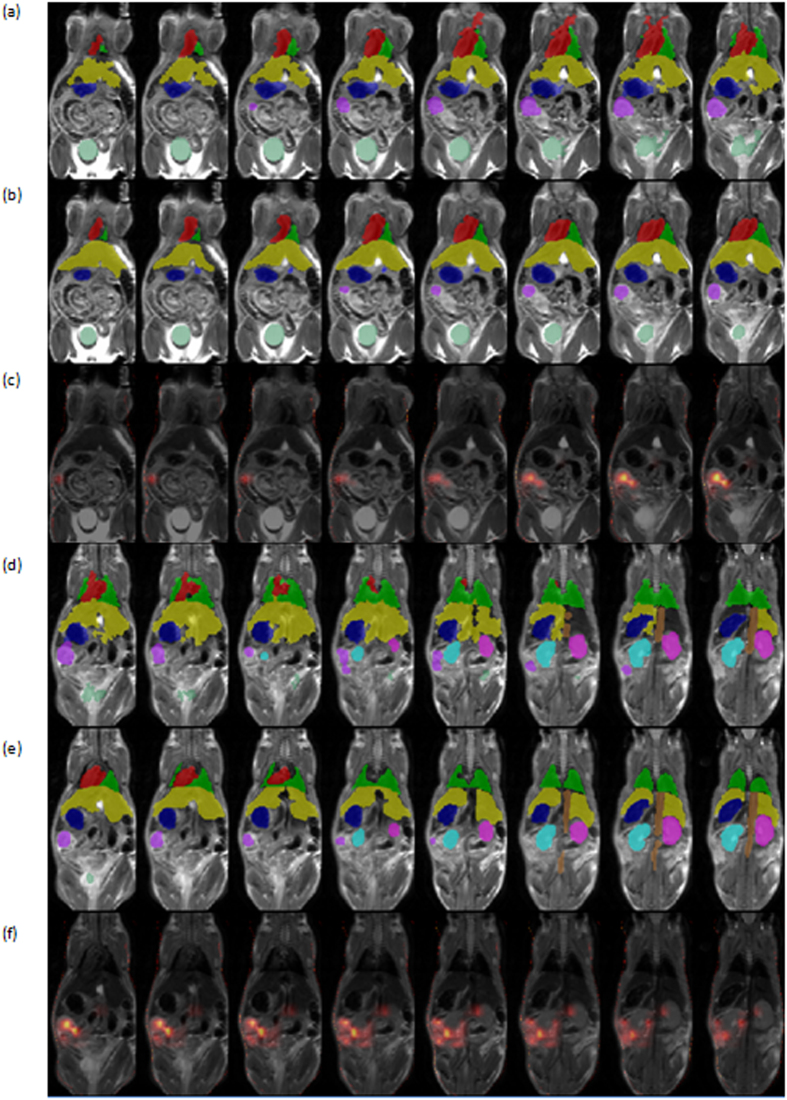
Illustration of segmentation results on internal organs. 16 axial slices are presents in the different columns, in two set of rows upper (**a–c**), lower (**d–f**). The organs presented are the ones used for calculating the dice score, namely heart (red), lungs (green), liver (yellow), stomach (blue), kidney left (cyan), kidney right (magenta), ovarian tumor (purple), bladder (dark green). Each set of rows includes: automatic segmentation (**a**,**d**) manual segmentation (**b**,**e**), aligned BLI images overlaid on the MR data (**c**,**f**).

The performance of the system is evaluated on twenty mice and nine organs including heart, lungs, liver, left kidney, right kidney, tumor, stomach, vena cava and bladder. To analyze the accuracy of the algorithm, dice coefficients of volume overlap were computed. Given two volumes automatic (A) and manual (M), S_A_, S_M_ represent the automatic segmentation and the manual ground truth segmentation respectively. Dice coefficients are computed as follows D = 2 * |S_A_∩S_M_|/|S_A_|+|S_M_|. Table 1 summarizes the average results obtained for the organs in terms of Dice coefficients compared to benchmarks results. Figure 7, demonstrates visualizations of results on 16 axial slices.

**Figure 8:**
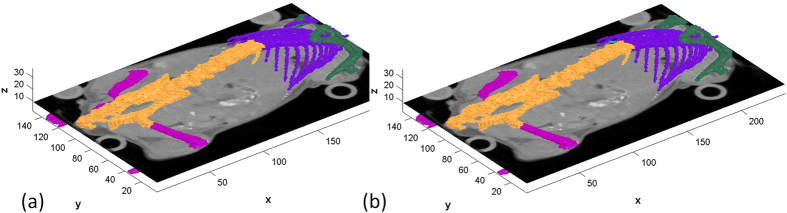
Examples of 3D skeleton segmentation results. Showing upper limbs (green), ribs (blue), sternum central (orange) and lower limbs (magenta). (**a**) Automatic segmentation (**b**). Manual segmentation.

CT images were acquired and aligned as described in Materials and Methods. Manual labeling to one of four classes (1) upper limb - including the scapula, humerus, radius, and ulna; (2) ribs; (3) sternum central - including the vertebrae and pelvis; and (4) lower limb - including the femur, fibula and tibia was performed as described in Materials and Methods. Table 2, presents results obtained on four skeleton structures with twenty mice by automatic segmentation of the CT modality alone (extracting scale = 7 of ~14 and without using the heatmap and kNN due to the CT high-contrast properties). Figure 8 shows visualizations of results in 3D.

### Computational complexity

Supervoxels are used to speed up the classifier and it is therefore important that they are generated efficiently. The computational cost for the supervoxel generation based on the multiscale pyramid is linear *O*(*n*) in the number of voxels in the image *n*. The classifier complexity is controlled by the kNN and SVM classification in each bounding box. The search time of the naive exact NN per test query is *O*(*dn*_*s*_) where *n*_*s*_ is the number of training supervoxel samples in each bounding box and *d* the number of features. For higher dimensions allowing approximate NN search with locality sensitive hashing^37^ can dramatically accelerate the algorithm. SVM training requires solving a quadratic problem and choosing the support vectors which is generally *O*(*dk*^2^) for the RBF kernel where k is the number of training points from kNN.

Our implementation on a standard Intel i7 CPU 3.07 GHz dual Core PC (with 2 GB RAM) takes less than 2 minutes for supervoxel pyramid generation on a multi modal set of 150 *×* 240 *×* 37 volumes of interest. This does not include the preprocessing to determine the correspondences to the selected MR channel (based on SPM and ITK software). Training and testing take less than one minute per mouse dataset and can be parallelized over datasets. The parameters of the algorithm were chosen empirically and include: pyramid parameters (e.g. fine, course coupling are *α* = 15,10 respectively, where the automatic multi-channel weight is explained in Materials and Methods, and the SVM-kNN classifier parameters k = 100, γ = 1).

## Discussion

*In-vivo* multi-modal imaging provides valuable complementary information in preclinical studies. Nevertheless advances in imaging techniques in preclinical research have not yet been matched by advances in computational methods enabling quantitative analysis. Despite the numerous segmentation approaches, segmentation of whole body small animal in multi-model imaging remains a difficult problem.

This study presents an automatic small animal imaging segmentation and learning method. Unlike previous approaches to whole body small animals, we do not rely on atlas registration. Instead, we incorporate an efficient multiscale approach to obtain supervoxels followed by a machine learning framework based on multiple SVM-kNN classifier each constrained to heatmap region, with high probability for the structure to obtain tissue segmentation. We demonstrate state-of-the-art accuracies on a large dataset, while segmenting more structures and being significantly more accurate than most other state of the art methods. It will be interesting to see the effect of deep learning strategies which have recently shown optimal results in many computer vision domains where large amounts of training data is available^48^. Finally, the robust and effective algorithm proposed can be adapted to a variety of segmentation scenarios and could have a large impact on this field.

## Additional Information

**How to cite this article**: Akselrod-Ballin, A. *et al*. Multimodal Correlative Preclinical Whole Body Imaging and Segmentation. *Sci. Rep.* **6**, 27940; doi: 10.1038/srep27940 (2016).

## Figures and Tables

**Table 1:** Summary of the results obtained compared to state of the art techniques for internal set of structures.

**Articles**	**Our System**	**Baiker** ***et al***.^10^	**Khemlinski** ***et al***.^5^	**Joshi** ***et al***.^7^	**Wang** ***et al***.^15^	**Xiao** ***et al***.^9^
#mice	20	15	3	1	14	13
Brain	–	0.76	–	0.8273	0.66 ± 0.13	–
Heart	0.81 ± 0.05	0.62	0.73 ± 0.08	0.8161	0.72 ± 0.05	–
Lungs	0.77 ± 0.04	0.65	0.46 ± 0.09	–	0.63 ± 0.14	–
Liver	0.75 ± 0.05	0.67	0.65 ± 0.03	0.5899	0.69 ± 0.05	–
Kidneys	0.77 ± 0.15	0.85	0.62 ± 0.17	–	0.69 ± 0.09	–
Tumor*	0.42 ± 0.23	–	–	–	–	–
Stomach	0.76 ± 0.09	–	–	–	–	–
Vena cava	0.55 ± 0.17	–	–	–	–	–
Bladder	0.71 ± 0.16	–	–	0.5481	–	–
Spleen	–	–	–	–	0.32 ± 0.1	–

Average Dice similarity coefficients are presented to compare between the automatic segmentation and the ground truth segmentation.

^*^Orthotopic ES2-Luc-DSRed Ovarian Carcinoma tumor xenograft.

**Table 2:** Summary of the results obtained for skeleton structures (Dice over entire set).

**Articles**	**Our System**	**Baiker** ***et al***.^9^	**Khemlinski** ***et al***.^5^	**Joshi** ***et al***.^7^	**Wang** ***et al***.^15^	**Xiao** ***et al***.^9^
#mice	20	15	3	1	14	13
Upper limbs	0.83 ± 0.12					
Ribs	0.83 ± 0.11					
Sternum central	0.92 ± 0.08					
Lower limbs	0.88 ± 0.09					
Skeleton		0.69			0.41 ± 0.06	0.17 **MAD

Equivalent quantitative results were not available from other studies.

^**^Both tables reported results in terms of Dice except for Xiao *et al*.^9^ reporting results in terms of the mean absolute distance (MAD) error.
